# Surgery

**Published:** 1920-09

**Authors:** J. Lacy Firth


### SURGERY.

**The Surgical Treatment of Gastric and Duodenal Ulcers.**— In our last issue the medical rather than the surgical treatment of the above diseases was considered. It seems appropriate, therefore, to continue discussion of the subject from the surgical point of view. A high degree of standardisation of the principles and methods of surgical treatment of duodenal ulcer has been reached, but in the more difficult problem of the treatment of gastric ulcer opinion is less settled. This is so because the comparatively simple and safe operation of gastro-enterostomy, in its present form, has proved highly successful for the former but often unsuccessful for the latter, for which, therefore, various other procedures are being practised and recommended, procedures which are for the most part of greater difficulty and danger.

Much light has been thrown on the whole subject by recent discussions, notably at the Royal Society of Medicine [1] and the Cambridge Annual Meeting of the British Medical Association, [2] and in several excellent papers, notably those by Dr. Hurst, [3] Mr. Sherren, [4] and Sir Berkeley Moynihan. [5]

The title of the last contribution on this list is "Disappointments after Gastro-enterostomy," and in it the author tabulates the causes of disappointment as follows :—

Firstly, those in which the operation has been performed in the absence of any organic lesion justifying it. In this class the errors have been primarily diagnostic, and such is the commonest cause of failure and explains nine out of ten cases. The conditions for which the operation was needlessly performed were (*a*) in functional disorders of the stomach or duodenum, and (*b*) in cases of chronic disease elsewhere. If the operation is performed for functional troubles, for example for atony or prolapse, often the patient's condition is made worse, and it may become the duty of the surgeon to undo the anastomosis. If no ulcer can be demonstrated on opening the abdomen gastro-enterostomy should not be performed, but a search should be made elsewhere for the cause of the symptoms which are present. Sir B. Moynihan has found that the operation has been performed wrongly for: (1) chronic appendicitis—in this disease there may be hæmatemesis or melæna ; (2) tuberculous disease of the intestine—with tuberculous disease of the cæcum there may be present such symptoms as pyloric spasm, hyperacidity, stomach pain, and vomiting ; (3) cholelithiasis or carcinoma of the gall-bladder—in the former "the pain of flatulence and of heaviness comes with fair regularity about half an hour after a meal," and there is often vomiting and acidity : "A very large proportion of the cases of cholelithiasis, at a time when no colic has occurred, are diagnosed as cases of gastric disease" ; (4) cirrhosis of the liver ; (5) splenic anæmia ; (6) tabes dorsalis with gastric crises ; (7) disseminated sclerosis, in one case in which the stomach became distended in the attacks of pain ; (8) the vomiting of pregnancy ; (9) lead poisoning ; (10) prolapse of the kidney ; (11) colic adhesions ; and (12) epigastric hernia.

Secondly, the failure of gastro-enterostomy may be due to the operation being incomplete in that some source of infection related to the ulcer has not been dealt with, for example cholelithiasis or chronic appendicitis. In duodenal ulcer the ulcer should be infolded. It will then disappear rapidly.

Thirdly, the failure is due to faults of technique, such as leaving a long jejunal loop, leaving the efferent loop kinked, rotating the jejunum round its longitudinal axis, making the opening too small, and not closing the opening made in the meso-colon.

Fourthly, late complications may develop, such as the formation of a jejunal ulcer or the change from simple to malignant ulceration in the stomach.

The foregoing enumeration of disappointments, the understanding of which is important, must not be allowed undue weight in estimating the value of gastro-enterostomy for suitable cases when skilfully performed, for they are very few in comparison with the generally excellent results of the operation and they are mostly avoidable. For the surgical treatment of chronic duodenal ulcer, which is four times as common as gastric ulcer, the standard procedure is still gastro-enterostomy, and the results are excellent. The same statement holds good for cases of cicatricial pyloric obstruction.

It is for chronic gastric ulcer that surgeons are more and more adopting other and move radical procedures, and to show how this change of practice has come about we must again review the opinions expressed by Sir B. Moynihan in his latest address on the subject at the Cambridge Meeting last June, and also the opinions of others who took part in the discussion.

Sir B. Moynihan stated that he had abandoned gastro-enterostomy for gastric ulcer for the following reasons. (*a*) The results were not so satisfactory as those following partial gastrectomy. The remaining morbidity was greater. (*b*) Cases returned with the ulcer still open. (*c*) Some few cases returned with carcinoma of the stomach after so long an interval that it was probable the cancerous change had occurred after the operation had been performed. (*d*) There is evidence that gastric ulcer has developed after gastro-enterostomy performed when the stomach was normal. Similarly Sir B. Moynihan has abandoned excision of ulcers after trial in 42 cases. He now "chooses partial gastrectomy whenever it can with reasonable safety be performed." The end of the stomach is applied to the side of the jejunum. The mortality in ten years has not been more than 2.5 per cent. For cases in which the patient is too feeble for gastrectomy or in which the ulceration is too advanced he chooses gastro-enterostomy in "Y," combined with jejunostomy. No food is given by the mouth for months or years, it is all given by the jejunostomy opening. With this treatment very large ulcers have been healed. The "Y" method of operating is that which most effectively prevents the biliary, pancreatic, and intestinal secretions gaining access to the stomach.

Mr. James Sherren has written: "Simple chronic gastric ulcers will heal as the result of gastro-jejunostomy correctly performed unless they are adherent and have perforated, their floor being formed of pancreas or liver." For the latter type of ulcer, owing to the failure of gastro-jejunostomy to cure, he has with increasing frequency performed partial gastrectomy, and up to December, 1919, had carried it out in 101 cases with 9 deaths. He believes that for gastric ulcer the operation of gastro-enterostomy will become less frequent than partial gastrectomy.

Mr. Rowlands [6] pertinently points out the dangers of these more radical procedures. At the Cambridge Meeting he said: "If partial gastrectomy becomes the most common operation for gastric ulcer, and is performed by a large number and variety of surgeons, the mortality will become very serious. Even in specially skilled hands it is about five times that of gastro-enterostomy."

We ourselves, nevertheless, anticipate that as experience grows and technique improves the results of partial gastrectomy will ultimately closely approach those of gastro-enterostomy in safety and general success. The history of most of the big innovations of surgery suggests this conclusion.

**The Physiological Results of Gastro-enterostomy.**—This subject has been discussed in most of the articles to which we have referred. Mr. Herbert J. Paterson's views as expressed at the Royal Society of Medicine in March and again at Cambridge are emphatic. He shows that there are two striking effects of the operation on the gastric contents, viz. (1) diminution of total acidity, and (2) increase of mineral chlorides. The diminution of acidity is due partly to the diminution of the total chlorides secreted by the stomach and partly to the neutralisation of free hydrochloric acid by the small quantity of bile and pancreatic juice which flows back into the stomach through the anastomosis. The increase of mineral chlorides is due to chlorides added from without, that is to the mineral chlorides of the bile and pancreatic juice. If entero-anastomosis ("Y" operation) is performed at the same time as gastro-enterostomy, the increase in mineral chlorides does not take place.

The conclusion is that gastro-jejunostomy is not a mechanical but a physiological operation. The operation "does not appreciably diminish the proportion of nitrogen or fat absorbed from food," and the patients do not suffer in nutrition.

Mr. James Sherren writes: "Gastro-jejunostomy acts by its influence on gastric motility and secretion. It secures a more rapid emptying of the stomach, and so prevents or counteracts pyloric spasm, and should abolish the excessive acidity which, with gastric stasis, Bolton has shown to be a cause of chronic ulceration." He has found that the greatest lowering of acidity is when the anastomosis is made toward the cardiac end of the stomach, and that it persists for years after the operation.

Sir B. Moynihan appears not to agree with the physiological views described above, maintaining that there is no proof that diminution of the normal acidity of the stomach contents favours the healing of gastric ulcers, and that hyperacidity is not the rule in gastric ulcer, being present in less than 30 per cent. of his own cases.

Dr. Arthur F. Hurst writes : " The more accurate investigations of Crohn by means of fractional test meals show that hypersecretion and hyperchlorhydria are constantly present " in gastric and duodenal ulceration. He concludes also from X-ray observations that three types of stomach exist. There is, firstly, a hypertonic type, which relatively quickly empties itself, and is a predisposing cause of duodenal ulceration in the presence of exciting causes, such as the production of toxins, mostly of bacterial origin, in other situations, *e.g.* from pyorrhœa alveolaris or chronic appendicitis. Secondly, there is a hypotonic type of stomach which is liable to develop gastric ulceration from similar causes. Thirdly, there is the isotonic normal stomach, which will not ulcerate even when the exciting causes are present.

We consider these speculations interesting and suggestive, and also that of Mr. G. E. Waugh,[7] who considers that the drag of a mobile ascending colon on the duodenum and pylorus injures the tissues there and leads to ulceration if the organs do not descend and relieve the tension.

In conclusion, we would direct attention to an excellent paper on the diagnosis of gastric ulcer by Sir B. Moynihan read at the opening session of the Harveian Society, October 23rd, 1919,[8] which we regret we have not space to review.

J. Lacy Firth.

### REFERENCES.

1 *Proc. Roy. Soc. Med.*, 1920, xiii., Sect. Surg., p. 105.

2 *Brit. M. J.*, 1920, ii. 99.

3 *Brit. M. J.*, 1920, i. 559.

4 *Lancet*, 1920, i. 691.

5 *Brit. M. J.*, 1919, ii. 33.

6 *Brit. M. J.*, 1920, ii. 107.

7 *Brit. J. Surg.*, 1920, vii. 343.

8 *Brit. M. J.*, 1919, ii. 765.

